# Analysis of Clinical Factors, Bacterial Genotyping, and Drug Resistance for Spinal Tuberculosis in South-Central China

**DOI:** 10.1155/2020/9871390

**Published:** 2020-01-22

**Authors:** Zheng Liu, Weiwei Li, Yilu Zhang, Yunqi Wu, Xiao Xiao, Zhicheng Sun, Yan Yang, Wenkai Hu, Xiyang Wang, Hao Zeng

**Affiliations:** ^1^Department of Spine Surgery, Xiangya Hospital, Central South University, 87#Xiangya Road, Changsha 410008, Hunan, China; ^2^Hunan Engineering Laboratory of Advanced Artificial Osteo-materials, Xiangya Hospital, Central South University, 87#Xiangya Road, Changsha 410008, Hunan, China; ^3^Department of Spinal Surgery, The First Affiliated Hospital of Guangxi Medical University, No. 6, Shuangyong Rd, Nanning, Guangxi 530021, China; ^4^Guangxi Key Laboratory of Regenerative Medicine, Nanning, Guangxi 530021, China

## Abstract

Spinal tuberculosis (STB), which is the most frequent and serious form of skeletal TB, is seriously harmful to a patient's life. However, very little research has been conducted on clinical isolates of STB. The purpose of this study was to genotype clinical isolates of *Mycobacterium tuberculosis* (MTB) from patients with STB, investigate their drug resistance profiles, and determine whether the genotypes and drug resistance patterns share any relationships with the demographic and clinical features of the patients. Preliminary species identification of the MTB strains was performed using a TCH/PNB culture method and multilocus polymerase chain reactions. Of the specimens collected from 85 hospital in-patients with STB at Xiangya Hospital, China, the 56 culture-positive MTB strains we identified were genotyped by spoligotyping. The strains were tested for resistance to anti-tuberculosis drugs (ATDs), and the demographic and clinical features of the patients were analyzed in combination with the genotyping and drug resistance results. Of the 56, cases, 53 involved *M. tuberculosis* and 3 involved *M. bovis*. Spoligotyping revealed 27 Beijing-type cases and 29 nonBeijing cases. When patients with STB were relapsing or experiencing systemic toxicity signs/symptoms (STS), the Beijing MTB-type strains predominated (*p* < 0.05), but when the patients were receiving initial treatment or lacked STS, the nonBeijing type MTB strains dominated. The Beijing and nonBeijing types differed in their resistance patterns to 8 ATDs, and the resistance rate of the Beijing type was higher than that of the nonBeijing type (*p* < 0.05). The bacteriological features of STB, including genotype and drug resistance, shared close relationships with the clinical features of patients with STB. Our data provide a reference for the diagnosis and treatment of STB.

## 1. Introduction

According to the latest report from the World Health Organization (WHO) in 2018 [1], the number of new cases of tuberculosis (TB) in the world in 2017 was about 889,000 and the estimated number of deaths was 388,000. As a developing country, China ranked the second among the 30 countries with high TB burdens in the world, and second only to India in the WHO report. Spinal tuberculosis (STB), which is the most frequent and serious form of skeletal TB, is seriously harmful to a patient's life. STB can cause vertebral-body fractures, spinal deformity, paraplegia, and even death [2–4]. It often also causes irreversible nerve damage and paralysis, a serious social and economic problem for those with it [5, 6]. Although surgical treatment of STB remains controversial, chemotherapy with anti-tuberculosis drugs (ATDs) is still required during surgical treatment of STB and is the most fundamental way to cure STB.

By the unremitting efforts of several generations, TB was once effectively controlled. But in recent years, with a large migratory and floating population, the prevalence of AIDS, unsatisfactory chemotherapy, and poor drug compliance in patients, TB has resurged to cause great harm to human societies. The increase in drug-resistant tuberculosis (DR-TB), especially multidrug-resistant tuberculosis (MDR-TB), has prompted people to pay more attention to drug treatment for TB [7, 8]. The serious problem of DR-TB in China, where the drug-resistance rate of *Mycobacterium tuberculosis *(MTB) at 46% accounts for a quarter of the total number of new drug-resistant patients in the world, has particularly alarmed the WHO [9, 10]. In recent years, the spread of MDR-TB and extensively drug-resistant tuberculosis (XDR-TB) has intensified the global spread of TB. The wide spread of drug-resistant bacteria has exacerbated its global presence, and the treatment and control of STB also faces a severe challenge.

STB develops from the blood-borne dissemination of MTB. Bacteriological diagnosis of STB is a most challenging task, one that is much more difficult than that for pulmonary tuberculosis (PTB). Thus, identifying drug resistance in STB isolates is vitally important for thorough treatment of such cases. Delayed initial diagnosis and confirmation of STB and the increasing incidence of multidrug-resistant STB in adults drives the need to improve the etiological diagnostics of STB, taking into account the biological and molecular properties of the pathogen [11, 12].

Owing to the development of various MTB typing methods, it is possible to make the epidemiology studies of TB be conducted. However, the special anatomical structure of the spine makes it difficult to obtain clinical specimens of STB except via surgery. Although the genetic polymorphisms of clinical isolates of MTB from PTB have been studied globally, very little research has been conducted on clinical isolates of STB [13].

Hence, the purpose of this study was to isolate MTB from specimens of STB lesions and genotype the MTB in them. We also analyzed the clinical features of the patients in combination with the genotyping and drug resistance data from the clinical isolates in an attempt to provide a preliminary reference tool for the diagnosis and treatment of STB.

## 2. Materials and Methods

### 2.1. Patients

Patients (86) diagnosed with STB who had received surgical treatment were selected from Xiangya Hospital, Central South University, China from June 2014 to June 2015. In all 86 cases, an intraoperative biopsy was obtained and the debrided material was sent for further histopathological examination. The debrided material from each patient had the appearance of a typical caseating granuloma. The collected specimens (surgical material from the lesions; namely, pus, granulation, and intervertebral disk and bone fragments) from the 86 patients were sent for MTB culture.

### 2.2. Culture and Identification of MTB Strains

**Figure 1:**
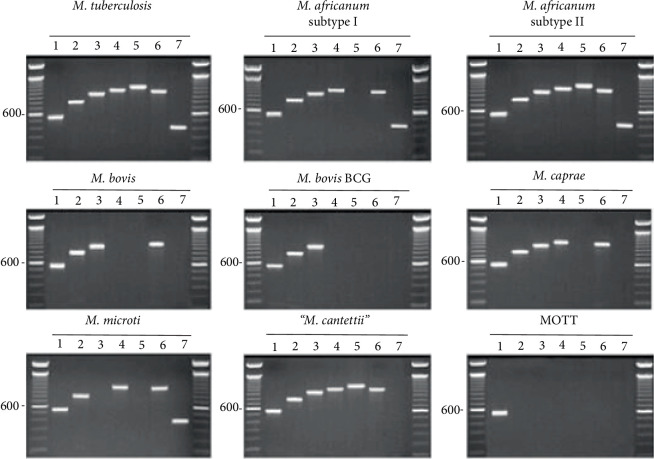
Result interpretation reference diagram for multilocus PCR.

According to the testing protocol for TB diagnosis in the National Laboratory [14], Lowenstein–Jensen (L–J) medium and the BACTEC^®^ MGIT^TM^ 960 system (Becton Dickinson, Sparks, Maryland, USA) were used for MTB cultivation and PNB/TCH medium were used for preliminary microbiological identification. The clinical specimens were processed, and each centrifuged sediment was inoculated into L–J medium or MGIT medium. The cultures were incubated at 37°C in 5% carbon dioxide for up to 8 weeks. The preliminary identification of MTB strains was performed using PNB/TCH medium. Multi-locus polymerase chain reaction (PCR) was used as a further identification method for MTB strains. We followed the method of Huard et al. [15] to PCR amplify 7 loci from a Mycobacterium Tuberculosis Complex (MTBC) strain as the PCR amplification targets (i.e., 16S rRNA, Rv0577, IS1561′, Rv1510, Rv1970, Rv3877/8, and Rv3120); these genes or gene fragments in the MTBC strains differ in their size distributions. Seven primer pairs, MTBC l–7, were selected to amplify the above-mentioned genes (Tables 1 and 2, Figure 1). Multi-locus PCR with these seven primer pairs was performed for all the specimens, which were stored as bacterial specimens from the MTB-positive cultures.

### 2.3. Spoligotyping and Spoligotype Analysis

**Figure 2:**
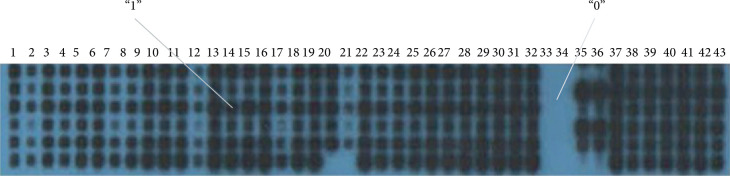
Spoligotyping fingerprints. Positive (interval existence) or negative (interval missing) hybridization results are represented by binary codes 1 and 0, respectively.

Spoligotyping was performed according to the methods used in previous studies [16–18]. Positive and negative hybridization results were represented here by the binary codes 1 and 0 (Figure 2), respectively, and analyzed using Microsoft Office Excel 2007 to group and order the hybridization patterns. The Excel file was incorporated into BioNumerics software for clustering analysis, and the Jaccard coefficient was used to analyze the data; this method divides identical or similar strains into one cluster. Spoligotypes common to more than one strain were designated as shared types and assigned a spoligotype international type number according to the updated version of the SpolDB4 international spoligotype database [19].

### 2.4. Drug Susceptibility Testing

Analysis of *M. tuberculosis* susceptibility to the first-line anti-TB drugs was done using the method of ratio according to “Guidelines for the implementation of Chinese tuberculosis control program (2008)” by the Chinese Ministry of Health. The first-line drugs tested were isoniazid [INH], rifampin [RIF], ethambutol [EMB], and streptomycin [SM], and the second-line drugs were ciprofloxacin [OPLX], ofloxacin [OFLX], capreomycin sulfate [CPM-SO4], and prothionamide [PTH].

### 2.5. Relationship Between Genotyping and Clinical Features

The spoligotyping results were combined with the clinical features of the patients with STB to further analyze the clinical significance of the genotyping results. The clinical features for STB were as follows: male/female, the spinal segments involved in STB, systemic toxicity signs/symptoms (STS), erythrocyte sedimentation rate (ESR), C-reaction protein (CRP), occupational environment, initial treatment and retreatment, history of TB, diabetes, and cardiovascular disease.

### 2.6. Statistical Analysis

SPSS 24 (SPSS, Inc., Chicago, IL) was used for the analysis. Independent sample *T* tests were used to test for drug resistance to different ATDs between Beijing and nonBeijing types. Chi-square tests were used to compare drug susceptibility between the Beijing and nonBeijing MTB strain types. When *p* was <0.05, the difference was considered to be statistically significant.

## 3. Results

Fifty-six specimens from 86 patients (65%) were culture positive for MTB. The clinical isolates from the 56 STB specimens were identified by TCH/PNB culturing. Fifty-three of the MTB strains grew in TCH and L–J medium but did not grow in PNB medium. Only 3 MTB specimens grew in L–J medium but did not grow in PNB/TCH medium. No MTB strains were able to grow in all three types of medium (PNB, TCH, L–J). Therefore, 53 cases of *M. tuberculosis *and 3 cases of *M. bovis *were preliminarily determined without mycobacterium other than tuberculosis (or *MOTT*). Moreover, the multi-locus PCR identification of the 53 cases of *M. tuberculosis *and 3 cases of *M. bovis* was consistent with the PNB/TCH/L–J medium identification results.

**Figure 3:**
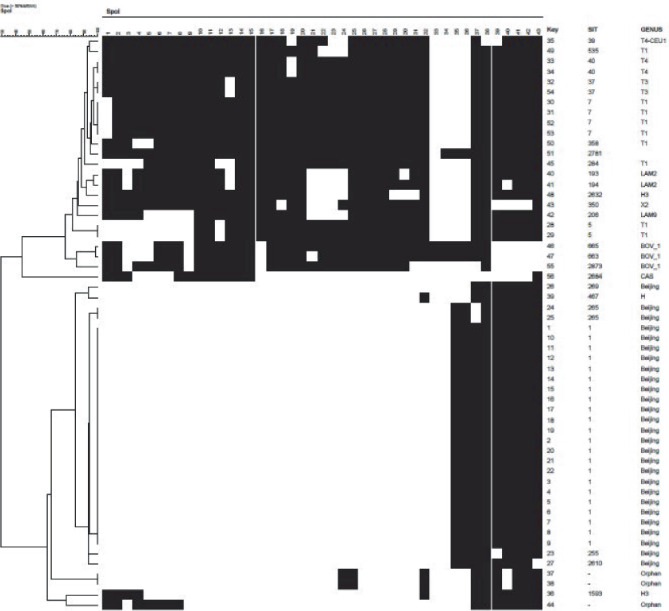
Cluster analysis diagram of the spoligotyping results for the 56 MTB strains.

Figure 3 and Table 3 show the spoligotypes of the 56 MTB strains. Of these, 27 strains (48.2%) were the Beijing type and 29 (51.8%) were the nonBeijing type, which included nine T1 type strains, two T3 and three T4, two LAM2 and one LAM9, three BOV_1, three Orphan, two H3, and one each of CAS, H, X2, and an unknown type.

Table 4 shows the different resistance levels of the MTB genotypes to different ATDs. Twenty-seven MTB Beijing-type strains displayed different resistance levels to 8 ATD types. The drug resistance rates from high to low were INH (17/27, 62.96%) < RFP (16/27, 59.26%) < SM (13/27, 48.15%) < EMB (9/27, 33.33%) < CPFX (5/27, 18.52%) < OFLX (4/27, 14.81%) < CPM-SO4 (3/27, 11.11%), and <PTH (1/27, 3.7%). Moreover, the number of strains with drug resistance to INH + RFP + SM and INH+RFP+SM+EMB was 10 (37.04%) and 8 (29.63%), respectively. The number of strains with MDR-MTB and XDR-MTB was 15 (55.56%) and 4 (14.81%), respectively. Twenty-nine strains of the nonBeijing MTB type displayed different resistance patterns to 8 ATD types. The rate of drug resistance from high to low was INH (4/29, 14.81%) < RFP (3/29, 11.11%) < SM (2/29, 6.89%) = EMB (2/29, 6.89%) = CPFX (2/29, 6.89%) < CPM-SO4 (1/29, 3.7%). All 29 strains were sensitive to OFLX and PTH. Resistance to INH + RFP + SM (6.89%) and MDR-MTB (6.89%) was seen in two strains each. Resistance to INH + RFP + SM + EMB or XDR-MTB was not observed in any strain.

Among the 27 Beijing-type strains, 3 (11.11%) were sensitive and 5 (18.52%) were typed as resistant to single-drugs, 15 (55.56%) were typed as MDR-MTB, and 4 (14.82%) were typed as XDR-MTB. Among the 29 non-Beijing-type strains, 20 (68.97%) were typed as sensitive, 7 (24.14%) were typed as single-drug resistant, 2 (6.9%) were typed as MDR-MTB, and there were no XDR-MTB-type strains. The drug resistance rate for the Beijing type was higher than that for the nonBeijing type (*p* < 0.05).

Table 5 shows the relationship between the genotyping data and the clinical features. A comparison between the Beijing and nonBeijing MTB strain types and the clinical features was performed. When patients with STB were in relapse or had active STS, the Beijing-type MTB strains formed the higher proportion (*p* < 0.05). When the patients with STB were undergoing their initial treatment or were without active STS, the nonBeijing MTB strain types formed the higher proportion.

Tables 6 and 7 show the drug resistance patterns for the situations where STB was accompanied with or without PTB, or with or without STS. In these situations, there were no significant differences in the drug resistance profiles of the Beijing MTB type (*p* < 0.05), whereas a significant difference in the drug resistance profiles of the nonBeijing MTB type was found in every situation (*p* < 0.05).

Table 8 shows the drug resistance profiles of the patients with STB during their initial treatment or retreatment. No significant difference in the drug resistance profiles of the nonBeijing MTB type (*p* < 0.05) was found, whereas a significant difference in the drug resistance profiles of the Beijing MTB type was found (*p* < 0.05).

## 4. Discussion

The special anatomy of the spine and its relatively bacteria-free environment make it difficult to study the etiology of STB, a rarely reported disease [13, 20, 21]. Therefore, we performed genotyping and drug resistance testing of clinical isolates from hospital patients with STB to better understand the bacterial features of STB. We also investigated the clinical characteristics of STB by testing for correlations between the bacterial features and the clinical aspects of STB.

Spoligotyping is currently one of the most widely used and most efficient molecular typing methods for MTB genotyping [19]. In 1995, Van Soolingen et al. [22] used spoligotyping for MTB genotyping in Beijing and its surrounding areas. These authors considered that the generation and spread of MTB strains within the Beijing family [23–25] may have resulted from the large number of Bacillus Calmette Guerin (BCG) long-term vaccinations that had taken place and antibiotics abuse.

Spoligotyping was used to genotype the 56 MTB strains in this study. The Beijing type and nonBeijing type accounted for 48.2% (27/56) and 51.8% (29/56) of the genotypes, respectively. Among the 27 Beijing-type strains, 85.2% (23/27) were the typical Beijing type, which positively hybridized only with 9 intervals between 35 and 43 bp. In contrast, 14.8% (4/27), belonging to the atypical Beijing family type, reacted negatively with 1 or 2 loci between 35–43 bp. The nonBeijing types were divisible into the following 12 genotypes: T1, T3, T4, LAM2, LAM9, BOV_1, CAS, H, H3, X2, Orphan, and Unclassified. Our results reveal the presence of substantial genetic polymorphisms in the MTB clinical isolates from patients with STB in Hunan province, of which the Beijing type accounted for 48.2% (27/56), a value much lower than for other Chinese regions for which there is data [23–26]. The percentage of the Beijing type in the MTB clinical isolates from PTB in northern China was higher than that in southern China (76.5% vs. 53.2%) [27]. The percentage of the nonBeijing type (51.8%) was higher than that of the Beijing type (48.2%) in this study, which differs from the data for PTB.

The spoligotyping data were combined with the clinical features of the STB patients to further analyze the clinical significance of the genotyping results. Although extrapulmonary TB infections in females seem to have occurred at twice the rate of that in the males, this observation was not statistically significant. These results are similar to those reported by Chen et al. [13]; that is, there is no significant difference in the male to female ratio of patients with STB.

The probability of contracting an STB infection in the thoracic and lumbar vertebrae is far greater than that for the cervical and sacral vertebrae. So we asked are some spinal segments more prone to infection with particular bacterial genotypes when patients are infected with MTB? Thus, we statistically analyzed the STB strains identified from the different vertebral segments and found that there was no significant difference in the type of MTB isolated from the spinal segments. The prevalence of STB differs in various vertebral segment locations, which probably relates to the different distribution of the anatomical structures, such as the vascular distribution, which will cause an uneven spinal distribution for MTB.

Along with improved living standards, immunity to TB has gradually increased, and the number of patients with obvious STS has decreased. Only 16 of the 86 patients that were assessed had STS (as shown by low fever, night sweats, emaciation and fatigue). Because the level of immunity to MTB in humans and STS share a relationship, could there be a correlation between STS and the type of MTB strain responsible for this condition? When seeking to answer this question, we found in all the 16 cases with STS that the MTB strains could be cultured successfully. By spoligotyping, the 16 strains were divided into 12 cases of the Beijing type (75%) and 4 of the nonbeijing type (25%). In the 40 cases without STS, there were 15 Beijing-type strains (37.5%) and 25 non-Beijing-type strains (62.5%). Therefore, no statistical differences in the types, with or without STS, were found. That is to say, the percentage of the Beijing type strains in the patients with STS was higher than the percentage of the nonBeijing type, which accounted for the majority genotype in patients without STS. Most studies have reported that Beijing-type strains are widely distributed and highly pathogenic [24, 25, 28]. This explains why most STB patients who were infected with the Beijing-type MTB presented with STS.

Increased ESR and elevated CRP values are often used to judge whether active TB exists in a person [29, 30]. Therefore, could an increased ESR or an elevated CRP level in patients with STB be linked with the MTB genotype? Table 6 shows that the increased ESR and CRP values for patients with STB bear no relationship with the MTB genotype.

STB relapse is a very serious problem for patients, in that it inflicts huge financial burdens and psychological pressures on patients and their families alike. The definition of relapse of STB in this work means that TB lesions are unhealed within 2 year after initial treatment, which need retreatment by surgery. In this study, the Beijing type accounted for 35% and nonBeijing type accounted for 65% of the genotypes in the initial treatment group. In contrast, the Beijing type accounted for 81.25% and the nonBeijing-type 18.75% of the two genotypes in the retreatment group. In other words, most patients in the retreatment group were infected with the Beijing type and most patients in the initial treatment group were infected with the nonBeijing type. In the retreatment group, most nonBeijing strains were the T type (6 in T1 and 2 in T4), which was the most numerous nonBeijing subtype. The Beijing-type and the nonBeijing T type comprised the most prevalent genotypes, and their strong pathogenicity may result in repeated disease outbreaks and a protracted course for STB, as this is linked with DR-TB.

This study provides useful data on the resistance levels of the different MTB genotypes to different ATDs. In China, in the fourth national epidemiological sampling survey of TB [31], the resistance rate for ATDs from high to low was as follows: INH (17.6%), SM (17.3%), RFP (16.6%), and EMB (1.5%). Contrastingly, the strains from the present study displayed the following resistance rates to ATDs: INH (37.5%), SM (26.79%), RFP (33.93%), and EMB (19.64%), the values of which are significantly higher than those from the fourth national epidemiological sampling survey of TB [31]. This highlights the serious nature of drug resistance for STB in this region of China. Some possible reasons for this are as follows. First, the damp and mild climate in Hunan Province is suitable for the growth of MTB, and the high incidence of TB and the high utilization rate of ATDs may have increased the resistance rate. Second, the data from the survey of the fourth national epidemiological sampling survey for TB were largely for PTB cases, which therefore cannot fully represent the situation for clinical isolates from patients with STB.

The present study found significant differences in the results of the drug resistance testing of the two genotypes. The resistance rate of the Beijing type was higher than that of the nonBeijing type. Therefore, strengthening the surveillance and treatment of Beijing type MTB will help with the prevention and control of epidemic TB in certain Chinese regions. The incidence MDR-TB and XDR-TB related to the Beijing type was higher than that seen for the nonBeijing type. However, the resistance rate to second-line ATDs was low for both genotypes. Therefore, when MDR-TB or XDR-TB is present, second-line ATDs should be tried.

In the Beijing type, the drug resistance rate was high with or without STS, but the rate was high with STS, while it was low without STS in the nonBeijing type. Hence, when STB patients do not have STS, rapid cultivation and genotyping of MTB should be performed to estimate an approximate resistance rate so that treatment can be delivered as soon as possible.

With rising drug resistance rates for MTB, relapsing STB is also becoming more prominent. In the nonBeijing type, the drug resistance rate was low irrespective of whether the patient was undergoing initial treatment or retreatment. However, the drug resistance rate during retreatment was higher than that during initial treatment for the Beijing type. Therefore, when patients present with STB relapse, rapid cultivation and genotyping of MTB should be performed to estimate an approximate resistance rate, in order to give timely treatment without waiting for all the drug resistance test results.

In summary, the bacteriological features of STB, including the genotypes responsible for it and their drug resistance profiles, were closely related with the clinical features associated with STB in the patients, and the present type of evaluation can provide a base-line reference for the diagnosis and treatment of STB.

There were some limitations in this study. The majority of patients lived in or around the Hunan area in south-central China. Therefore, our data and results might only be representative of this region. Because the sample size in this study was relatively small, additional studies with more participants are needed to confirm our results.

## Figures and Tables

**Table 1:** Seven primer pairs used in multilocus PCR.

Gene	Primer name	Primers (5′→3′)		Fragment size (bp)
16S rRNA	16SRNAF	ACGGTGGGTACTAGGTGTGGGTTTC	543	
	16SRNAR	TCTGCGTTACTAGCGACTCCGACTTCA		
Rv0577	Rv0577F	ATGCCCAAGAGAAGCGAATACAGGAA	786	
	Rv0577R	CTATTGCTGCGGTGCGGGCTTCAA		
IS1561′	IS1561F	GCTGGGTGGGCCCTGGATACGTGAACTCT	943	
(Rv3349c)	IS1561R	AACTGCTCACCCTGGCACCACCATTGACT		
Rv1510	Rv1510F	GTGCGCTCCACCCAAATAGTTGC	1033	
	Rv1510R	TGTCGACCTGGGGCACAAATCAGTC		
Rv1970	Rv1970F	GCGCAGCTGCCGGATGTCAAC	1116	
	Rv1970R	CGCCGGCAGCCTCACGAAATG		
Rv3877/8	Rv3877/8F	CGACGGGTCTGACGGCCAAACTCATC	999	
	Rv3877/8R	CTTGCTCGGTGGCCGGTTTTTCAGC		
Rv3120	Rv3120F	GTCGGCGATAGACCATGAGTCCGTCTCCAT	404	
	Rv3120R	GCGAAAAGTGGGCGGATGCCAGAATAGT		

**Table 2:** Algorithm to identify individual MTBC subspecies.

Organism(s)				Results				Profile
	16S	Rv0577	IS1561′	Rv1510	Rv1970	Rv3877/8	Rv3120	
	rRNA(1)	(2)	(3)	(4)	(5)	(6)	(7)	
*M. tuberculosis*	**+**	**+**	**+**	**+**	**+**	**+**	**+**	1234567
*M. africanum subtype II*	**+**	**+**	**+**	**+**	**+**	**+**	**+**	1234567
*M. canettii*	**+**	**+**	**+**	**+**	**+**	**+**	−	123456
*M. africanum subtype I*	**+**	**+**	**+**	**+**	−	**+**	**+**	123467
*M. caprae*	**+**	**+**	**+**	**+**	−	**+**	−	12346
*M.bovis*	**+**	**+**	**+**	**−**	−	**+**	−	1236
*M. bovis BCG*	**+**	**+**	**+**	−	−	−	−	123
*M. microti*	**+**	**+**	−	**+**	**−**	**+**	**+**	12467
*MOTT*	**+**	−	−	−	−	−	−	1

**Table 3:** Results of spoligotyping.

Genotype family	Cases of strain	SIT (strain)
Beijing	27	1 (22), 269 (1), 265 (2), 255 (1), 2610 (1)
Nonbeijing	29	
T1	9	7 (4), 535 (1), 284 (1), 358 (1), 5 (2)
T3	2	37 (2)
T4	3	40 (2)
LAM2	2	193 (1), 194 (1)
LAM9	1	206 (1)
BOV_1	3	663 (1), 665 (1), 2873 (1)
CAS	1	2684(1)
H	1	467 (1)
H3	2	1593 (1), 2632 (1)
X2	1	350 (1)
Orphan	3	−
Unclassified	1	2781 (1)

SIT: Spoligotype International Type.

**Table 4:** Analysis of different MTB genotypes resistance to different ATDs (resistance rate: %).

Drug species	Beijing type^∗^		Nonbeijing type		All strains	
	Drug-resistant strains	Resistance rate	Drug-resistant strains	Resistance rate	Drug-resistant strains	Resistance rate
INH	17	62.96	4	14.81	21	37.5
RFP	16	59.26	3	11.11	19	33.93
SM	13	48.15	2	6.89	15	26.79
EMB	9	33.33	2	6.89	11	19.64
CPM-SO4	3	11.11	1	3.70	4	7.14
CPFX	5	18.52	2	6.89	7	12.50
OFLX	4	14.81	0	0	4	7.14
PTH	1	3.70	0	0	1	1.79
INH + RFP + SM	10	37.04	2	6.89	12	21.43
INH + RFP + SM + EMB	8	29.63	0	0	8	14.29
MDR	15	55.56	2	6.89	17	30.36
XDR	4	14.81	0	0	4	7.14

^∗^Independent sample *T* test of drug resistance to different ATDs between Beijing and nonBeijing types, *p* < 0.05.

**Table 5:** Relationship between genotyping and clinical factors.

Clinical factors		Strain*s*	Beijing type	Nonbeijing type	Chi-square value/*p*-value
Gender	Male	29	17	12	2.609/0.106
	Female	27	10	17	
Infected spinal segment	Cervical	6	2	4	2.025/0.567
	Thoracic	20	9	11	
	Lumbar	22	13	9	
	Sacral	8	3	5	
STS	With	16	12	4	6.437/0.011
	Without	40	15	25	
ESR	Rise	38	20	18	0.924/0.399
	Normal	18	7	11	
CRP	Rise	42	18	24	1.931/0.221
	Normal	14	9	5	
Occupation environment	City	15	6	9	0.554/0.552
	Country	41	21	20	
Treatment	Initial treatment	40	14	26	9.791/0.003
	Retreatment	16	13	3	
PTB	With	6	2	4	0.695/0.671
	Without	50	25	25	
History of diabetes	With	12	4	8	1.355/0.334
	Without	44	23	21	
History of cardiovascular disease	With	11	7	4	1.304/0.322
	Without	45	20	25	

**Table 6:** Drug resistance in STB accompanied with or without PTB.

Drug resistance	Without PTB			STB accompanied with PTB		
	Beijing type^∗^	Nonbeijing type^#^	Total	Beijing type	Nonbeijing type	Total
Sensitive	3	20	23	0	0	0	
Single-drug resistant	5	5	10	0	2	2	
Multi-drug resistant	14	0	14	1	2	3	
Extensively drug resistant	3	0	3	1	0	1	
Total	25	25	50	2	4	6	

^∗^Chi-square test, compare with the group accompanied with PTB, *p* < 0.05.

^#^Chi-square test, compare with the group accompanied with PTB, *p* < 0.05.

**Table 7:** Drug resistance in STB accompanied with or without STS.

Drug resistance	Accompanied with STS			Without STS		
	Beijing type^∗^	Nonbeijing type^#^	Total	Beijing type	Nonbeijing type	Total
Sensitive	0	0	0	3	20	23	
Single-drug resistant	2	2	4	3	5	8	
Multi-drug resistant	8	2	10	7	0	7	
Extensively drug resistant	2	0	2	2	0	2	
Total	12	4	16	15	25	40	

^∗^Chi-square test, compare with the group without STS, *p* < 0.05.

^#^Chi-square test, compare with the group without STS, *p* < 0.05.

**Table 8:** Drug resistance in STB in initial treatment or retreatment.

Drug resistance	In initial treatment			In retreatment		
	Beijing type^∗^	Nonbeijing type ^#^	Total	Beijing type	Nonbeijing type	Total
Sensitive	3	19	22	0	1	1
Single-drug resistant	4	6	10	1	1	2
Multi-drug resistant	7	1	8	8	1	9
Extensively drug resistant	0	0	0	4	0	4
Total	14	26	40	13	3	16

^∗^Chi-square test, compare with the group in retreatment, *p* < 0.05.

^#^Chi- square test, compare with the group in retreatment, *p* > 0.05.

## Data Availability

The data used to support the findings of this study are included within the article.

## References

[1] WHO Global tuberculosis report 2018.

[2] Wang L. J., Zhang H. Q., Tang M. X., Gao Q. L., Zhou Z. H., Yin X. H. (2017). Comparison of three surgical approaches for thoracic spinal tuberculosis in adult: minimum 5-year follow up. *Spine*.

[3] Zeng H., Shen X., Luo C. (2016). 360-degree cervical spinal arthrodesis for treatment of pediatric cervical spinal tuberculosis with kyphosis. *BMC Musculoskeletal Disorders*.

[4] Zhang P., Wei P., Wang X. (2016). Minimum 5-year follow-up outcomes for single-stage transpedicular debridement, posterior instrumentation and fusion in the management of thoracic and thoracolumbar spinal tuberculosis in adults. *British Journal of Neurosurgery*.

[5] Pigrau-Serrallach C., Rodrã­-Guez-Pardo D. (2013). Bone and joint tuberculosis. *European Spine Journal*.

[6] Liu Z., Wang X., Xu Z. (2016). Two approaches for treating upper thoracic spinal tuberculosis with neurological deficits in the elderly: a retrospective case-control study. *Clinical Neurology & Neurosurgery*.

[7] Held M., Laubscher M., Zar H. J., Dunn R. N. (2014). GeneXpert polymerase chain reaction for spinal tuberculosis: an accurate and rapid diagnostic test. *The Bone & Joint Journal*.

[8] Li L., Zhang Z., Luo F. (2012). Management of drug-resistant spinal tuberculosis with a combination of surgery and individualised chemotherapy: a retrospective analysis of thirty-five patients. *International Orthopaedics*.

[9] Guglielmetti L., Jaspard M., Le Dû D. (2016). Long-term outcome and safety of prolonged bedaquiline treatment for multidrug-resistant tuberculosis. *European Respiratory Journal*.

[10] Judge D., Krause V. (2016). Multidrug-resistant tuberculosis in the northern territory: a 10-year retrospective case series. *Communicable Diseases Intelligence Quarterly Report*.

[11] Raj A., Singh N., Gupta K. B. (2016). Comparative evaluation of several gene targets for designing a multiplex-PCR for an early diagnosis of extrapulmonary tuberculosis. *Yonsei Medical Journal*.

[12] Lima J. F., Guedes G. M., Lima J. F. (2015). Single-tube nested PCR assay with in-house DNA extraction for *Mycobacterium tuberculosis* detection in blood and urine. *Journal of the Brazilian Society of Tropical Medicine*.

[13] Chen S. T., Zhao L. P., Dong W. J. (2014). The Clinical features and bacteriological characterizations of bone and joint tuberculosis in China. *Scientific reports*.

[14] Association BPCoCA (2006). *Laboratory Procedures for Diagnosis of Tuberculosis*.

[15] Huard R. C., Lazzarini L. C., Butler W. R., Van S. D., Ho J. L. (2003). PCR-based method to differentiate the subspecies of the *Mycobacterium tuberculosis* complex on the basis of genomic deletions. *Journal of Clinical Microbiology*.

[16] Kamerbeek J., Schouls L., Kolk A. (1997). Simultaneous detection and strain differentiation of *Mycobacterium tuberculosis* for diagnosis and epidemiology. *Journal of Clinical Microbiology*.

[17] Dong H. Y., Lu B., Zhang Y. Y. (2009). Introduction to the standard operation program of spoligotyping on *Mycobacterium tuberculosis* in China. *Chinese Journal of Epidemiology*.

[18] Groenen P. M. A., Bunschoten A. E., Soolingen D. V., Errtbden J. D. A. V. (1993). Nature of DNA polymorphism in the direct repeat cluster of *Mycobacterium tuberculosis*; application for strain differentiation by a novel typing method. *Molecular Microbiology*.

[19] Brudey K., Driscoll J. R., Rigouts L. (2006). *Mycobacterium tuberculosis* complex genetic diversity: mining the fourth international spoligotyping database (SpolDB4) for classification, population genetics and epidemiology. *BMC Microbiology*.

[20] Vyazovaya A., Mokrousov I., Solovieva N. (2015). Tuberculous spondylitis in Russia and prominent role of multidrug-resistant clone *Mycobacterium tuberculosis* Beijing B0/W148. *Antimicrobial Agents & Chemotherapy*.

[21] Weng C. Y., Ho C. M., Dou H. Y. (2013). Molecular typing of *Mycobacterium tuberculosis* isolated from adult patients with tubercular spondylitis. *Journal of Microbiology, Immunology and Infection*.

[22] Soolingen D. V., Qian L., Haas P. E. D. (1995). Predominance of a single genotype of *Mycobacterium tuberculosis* in countries of East Asia. *Journal of Clinical Microbiology*.

[23] Li S., Min Y., Pourcel C., Peng Z., Zhu C. M. (2007). Genotyping study of 216 *Mycobacterium tuberculosis* strains isolated from the patients in tibet with MLVA and spoligotyping. *Chinese Journal of Microbiology & Immunology*.

[24] Deng J. P., Liu H. C., Wang B., Begna T. (2015). Spoligotyping of *Mycobacterium tuberculosis* in the south of Sichuan province. *Chinese Journal of Zoonoses*.

[25] Liu F., Liu Z., Wang X., Deng J. P., Liu H. C. (2007). Genotyping study of 208 *Mycobacterium tuberculosis* clinical isolates from guangxi with spoligotyping. *Chinese Journal of Zoonoses*.

[26] Cao X. H., Liu Z. G., Zhao X. Q., Bing L. U., Zhang Y. Y., Wan K. L. (2008). Genotyping analysis of 220 clinical isolates of *Mycobacterium tuberculosis* from Beijing area. *Chinese Journal of Zoonoses*.

[27] Pang Y., Zhao Y. L., Zhou Y. (2012). Spoligotyping and drug resistance analysis of *Mycobacterium tuberculosis* strains from national survey in China. *PLoS One*.

[28] Zhang Y. Y., Huang M. X., Zhao X. Q., Zhang L. S., Liu Z. G., Wan K. L. (2011). Rapid identification of *Mycobacterium bovis* BCG with spoligotyping and multi-locus PCR. *Chinese Journal of Zoonoses*.

[29] Liu Z., Wang J., Chen G.-Z. (2019). Clinical characteristics of 1378 inpatients with spinal tuberculosis in general hospitals in south-central China. *BioMed Research International*.

[30] Liu Z., Zhang P., Zeng H., Xu Z., Wang X. (2018). A comparative study of single-stage transpedicular debridement, fusion, and posterior long-segment versus short-segment fixation for the treatment of thoracolumbar spinal tuberculosis in adults: minimum five year follow-up outcomes. *International Orthopaedics*.

[31] Liu Y., Jiang G., Zhao L., Xu Z., Wang X. (2002). Drug resistance of *Mycobacterium tuberculosis* in a nationwide epidemiological survey in China in the year of 2000. *Chinese Journal of Tuberculosis and Respiration*.

